# Alteration of the Donor/Acceptor Spectrum of the (*S*)-Amine Transaminase from *Vibrio fluvialis*

**DOI:** 10.3390/ijms161126007

**Published:** 2015-11-11

**Authors:** Maika Genz, Clare Vickers, Tom van den Bergh, Henk-Jan Joosten, Mark Dörr, Matthias Höhne, Uwe T. Bornscheuer

**Affiliations:** 1 Department of Biotechnology and Enzyme Catalysis, Institute of Biochemistry, University of Greifswald, 17487 Greifswald, Germany; maika.genz@uni-greifswald.de (M.G.); clare.vickers@uni-greifswald.de (C.V.); mark.doerr@uni-greifswald.de (M.D.); 2 Bio-Prodict, Nieuwe Marktstraat 54E, 6511 AA Nijmegen, The Netherlands; tvandenbergh@bio-prodict.nl (T.B.); joosten@bio-prodict.nl (H.-J.J.); 3 Protein Biochemistry, Institute of Biochemistry, University of Greifswald, 17487 Greifswald, Germany; matthias.hoehne@uni-greifswald.de

**Keywords:** amine transaminase, *Vibrio fluvialis*, amine, protein design, library creation

## Abstract

To alter the amine donor/acceptor spectrum of an (*S*)-selective amine transaminase (ATA), a library based on the *Vibrio fluvialis* ATA targeting four residues close to the active site (L56, W57, R415 and L417) was created. A 3DM-derived alignment comprising fold class I pyridoxal-5′-phosphate (PLP)-dependent enzymes allowed identification of positions, which were assumed to determine substrate specificity. These positions were targeted for mutagenesis with a focused alphabet of hydrophobic amino acids to convert an amine:α-keto acid transferase into an amine:aldehyde transferase. Screening of 1200 variants revealed three hits, which showed a shifted amine donor/acceptor spectrum towards aliphatic aldehydes (mainly pentanal), as well as an altered pH profile. Interestingly, all three hits, although found independently, contained the same mutation R415L and additional W57F and L417V substitutions.

## 1. Introduction

Chiral amines are highly versatile building blocks for the pharmaceutical and agrochemical industry. The biocatalytic asymmetric synthesis of amines can be achieved using amine transaminases (ATA), which are therefore currently widely investigated. The advantages of using ATAs compared to standard organo- or metallo-catalysis are increased process efficiency by the one-step transamination and, additionally, the avoidance of toxic heavy metals and organic solvents [1]. Although naturally-occurring enzymes rarely offer ideal manufacturing catalysts, protein engineering provides an effective technique to overcome this limitation. In the last decade, the development of sophisticated experimental and computational protein engineering tools enabled scientists to reach the third wave of biocatalysis [2]. The combination of different protein engineering strategies led to the development of enzymes as highly effective biocatalysts, which are already used in various large-scale industrial applications. The best example for the enzymatic synthesis of amines is the industrial production of the drug sitagliptin by using an engineered (*R*)-amine transaminase [3]. By extensive protein engineering, this (*R*)-ATA was modified with 27 amino acid changes to suit the requirements for the industrial process.

Most of the transaminases (TA) naturally occur as dimers. There are some examples where the biological unit is defined as a tetramer, e.g., the TA from *Arthrobacter aurescens* TC1 [4]. Nevertheless, the active site of all TAs is located at the dimer interface, resulting in two active sites per dimer. The active site therefore is built from amino acid residues coming from both monomers. Transaminases contain the cofactor pyridoxal-5′-phosphate (PLP) and can be found in the PLP-dependent fold types I and IV. In all TAs of fold class I, the active site is composed of two binding pockets: the large and the small binding pocket. The large binding pocket recognizes the α-carboxylate function of the substrates and is located above the 3′-oxygen of the PLP. The small binding pocket next to the phosphate group of PLP provides space for the hydrophobic side chain of aromatic amino acids or, alternatively, the acidic side chain of the acidic substrate, which can be bound in the same pocket. This is realized by a flexible arginine residue (flipping arginine). This arginine either forms a salt bridge to the carboxylate group or moves out of the active site.

The arrangement of the active site, especially the interplay of residues contributed from both monomer units, makes rational design and/or protein engineering approaches on transaminases much more challenging. Nevertheless, there have been some examples where ATAs have been successfully engineered [5,6,7]. In previous studies, we found that the ATA from *Vibrio fluvialis* (VF-ATA) is a good template for protein engineering, as this enzyme seems to tolerate mutations in both the small and the large binding pocket [6,7]. Using this VF-ATA, we identified two mutants (F85L/V153A and Y150F/V153A) showing 30-fold increased activity in the conversion of (*S*)-phenylbutylamine and (*R*)-phenylglycinol, respectively [6]. Additionally, we could ensure access to optically-active 3-alkyl-substituted chiral amines by combining an enoate reductase and the VF-ATA in a cascade reaction. Initially, the VF-ATA wild-type showed only modest enantioselectivity (14% diastereomeric excess (de)) in the amination. With the help of protein engineering, we created two variants with substantially improved diastereoselectivities: variant Leu56Val exhibited a higher (*R*)-selectivity (66% de), whereas the Leu56Ile substitution caused a switch in enantiopreference to furnish the (*S*)-configured diastereomer (70% de) [7].

The goal of this study was to alter the donor/acceptor spectrum of VF-ATA, as the wild-type enzyme has a strong preference only for pyruvate and a few other α-keto acids and their corresponding α-amino acids. Furthermore, ATAs accepting aldehydes as amino acceptors would be useful to make primary amines serving as precursors in polymer synthesis, such as ω-amino carboxylic acids [8,9].

Several tools are available for protein engineering, such as directed evolution (*i.e.*, random mutagenesis in combination with high-throughput screening) or rational protein design based on protein structures in combination with computer modeling. For the rational design of a “small, but smart” library, we used the commercially available 3DM software [10]. 3DM is a protein superfamily analysis suite, which uses highly sophisticated algorithms for the calculation of alignments based on structure/sequence-function relationships of a defined superfamily [11]. A database on the PLP-dependent transferases of fold type I was used, which contains 725 structures on which 61,560 sequences were aligned [12]. Within this superfamily database, a sub-dataset based on the target protein, the amine transaminase from *Vibrio fluvialis* (PDB-Id: 4E3Q [5]) was created with 12,956 aligned sequences. In this sub-database, a core sequence mainly based on structural characteristics is defined with a unique 3DM numbering system. 3DM generates structure alignments based on the monomers of all available structure files. Combined with data extracted for the sequences in the alignment, such as mutation data from the literature and correlated mutation scores, these alignments reveal amino acid functions. 3DM also extracts contact data (e.g., dimerization data, substrate contacts, *etc.*) from all of the available PDB files. As all of these different data types in 3DM are stored with 3D localization information (e.g., the 3D numbers), all of the different data types can be compared to each other and directly visualized in any of the 3D structures. These features make 3DM an excellent platform for the analysis of active sites that are composed of multiple monomers.

**Figure 1:**
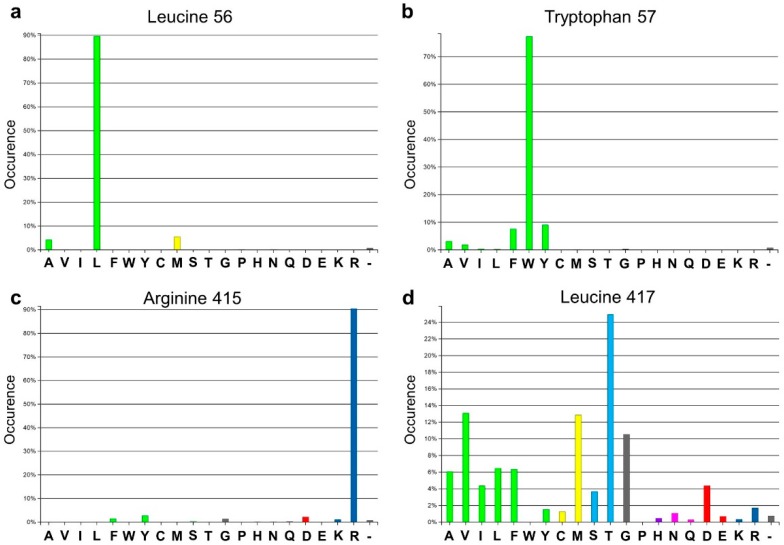
Amino acid distribution of the four target sites in the *Vibrio fluvialis* amine transaminase (VF-ATA) library. The amino acid distribution is based on the sub-dataset created by 3DM. (**a**) Amino acid distribution for leucine 56 (3DM No. 46); (**b**) amino acid distribution for tryptophan 57 (3DM No. 47); (**c**) amino acid distribution for arginine 415 (3DM No. 346); (**d**) amino acid distribution for leucine 417 (3DM No. 348). Colors define amino acids with the same or different physicochemical properties, respectively.

Analysis of the active site of the VF-ATA highlighted four residues in the large binding pocket, Leu56, Trp57, Arg415 and Leu417, which appear to have a sterically limiting role in the active pocket and, thus, potentially limit the substrate scope. Additionally, 3DM analysis showed that three positions out of the four chosen ones are highly conserved amongst amine:pyruvate transaminases (Figure 1). At position 56 (3DM No. 46), leucine is conserved with 89.7%; at position 57 (3DM No. 47), tryptophan is represented with 77.3%; and at position 415 (3DM No. 346), arginine is conserved with 90.5%. This implicates, that these three residues play a significant role in substrate recognition. Thus, including these in the target set may identify protein variants, which have a non-natural substrate scope. According to the hydrophobic nature and strong conservation of leucine 56 and tryptophan 57, an alphabet encoding for hydrophobic amino acids was chosen to retain the hydrophobicity at these position and, thereby, the hydrophobic network. We assumed that keeping the hydrophobic network results in more active protein variants by preventing the damage of the active site, but still results in proteins with new properties, as non-conserved amino acids will be introduced. Due to its important position in the dual substrate recognition of TAs, the flipping arginine of the VF-ATA (arginine 415) was also targeted to change it into hydrophobic amino acids. Additionally, position 417 (3DM No. 348) was included in the library, where the most common amino acid is threonine with 24.9% conservation in the sub-dataset of ATAs. This library was then created and analyzed by high-throughput screening to identify novel variants with the desired altered characteristics.

## 2. Results and Discussion

### 2.1. Design and Creation of the Library

**Figure 2:**
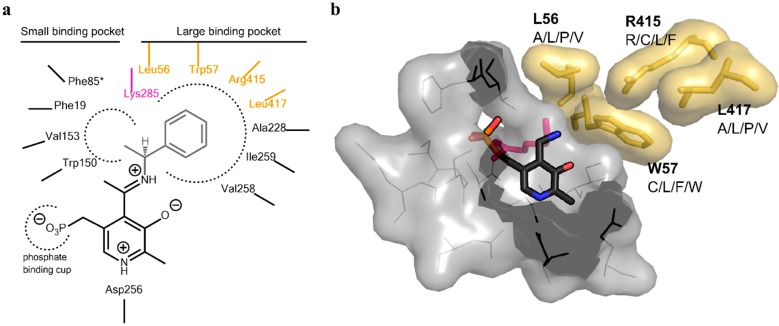
Active site architecture of the ATA from *Vibrio fluvialis* (PDB code: 4E3Q [5]). (**a**) Schematic drawing of the large and small binding pocket around the external aldimine intermediate (pyridoxal-5′-phosphate (PLP)-Schiff base with (*S*)-phenylethylamine). Amino acids covered in the library are highlighted in yellow, the catalytic lysine is highlighted in purple; (**b**) View of the substrate binding pocket. The pyridoxamine-5’-phosphate (PMP) is shown in sticks (dark grey, colored by element). The four residues on which the library was based are displayed together with the amino acid substitutions identified for the mutant library (yellow sticks including van der Waals radius as the surface). The catalytic lysine can be seen in the background behind PMP (purple sticks). The PMP binding pocket is displayed as a light gray surface, including the amino acids shown as lines.

Inspection of the active site of the VF-ATA highlighted four residues in the substrate binding pocket, which could have a steric influence on the change of the amine donor/acceptor spectrum (Figure 1 and Figure 2). Due to synergistic effects, it was assumed that single mutations would only produce small improvements in the change of the donor/acceptor spectrum. Thus, a library containing at least one up to four changes in the resulting amino acid sequence was designed. An amino acid alphabet was selected that included both large and small hydrophobic amino acids at each target site. Thus, the effect on the pocket volume would range from subtle (variants with mutations to both large and small amino acids resulting in a similar pocket volume, but increased hydrophobicity compared to the wild-type (wt)) to dramatic changes (variants with mutations to all small amino acids at the target sites resulting in a much increased pocket volume). Next, a “small, but smart” library was created containing the following changes encoded for the desired positions: A/L/P/V at residue 56, C/L/F/W at residue 57, R/C/L/F at residue 415 and A/L/P/V at residue 417. A library containing these changes was prepared consecutively by a two-step site-directed mutagenesis approach, as the degenerate primer pairs covered each of two sites (L56 + W57 and R415 + L417). The correctness and quality of the library was verified by sequencing.

### 2.2. Screening of the Library with an Automated Robotic Platform

The degenerate variation at the four sites of VF-ATA, which are located in the large binding pocket (L56, W57, R415 and L417) yields theoretically 256 mutants. To ensure >95% coverage of the library, 1120 colonies (16 plates, 96-well microtiter format) were screened for activity against the amine donor (*S*)-1-phenylbutylamine ((*S*)-PBA) and the amino acceptors pyruvate and pentanal, especially as the library contained the variation of the flipping arginine (R415) to smaller hydrophobic side chains [13,14]. Screening of the library was realized via our automatic robotic platform [15,16].

The activity of all variants from the 16 plates was determined using a modified acetophenone-based assay with spectrophotometric measurement of the aromatic ketone formed at 242 nm [17]. Each plate was inspected, and reaction rates were compared to the wild-type (wt) activity (wt VF-ATA served as positive control on each plate). Six hits were found to have a higher activity than the wild-type using pentanal, and no hits with higher activity than the wt VF-ATA could be identified using pyruvate as the amino acceptor. All hits were analyzed by sequencing, expressed on a larger scale, purified via their His-tag using Ni-NTA and verified for activity against pentanal or pyruvate with (*S*)-PBA as the amine donor (Table 1).

In all cases except variant M5 (L56V/W57F), the flipping arginine had been mutated to either leucine (M1–3), cysteine (M4) or phenylalanine (M6). None of the five variants showed any activity towards pyruvate, but an increase in their activity towards pentanal (except variant M6, Table 1). There are two surprising findings in this initial analysis of the hits from the robotic screening. First of all, it is noticed that the change of the flipping arginine to phenylalanine in variant M6 (R415F/L417A) does not reveal a significant increase in the activity against pentanal. Secondly, variant M5 (L56V/W57F), where the flipping arginine is still preserved, showed a slight improvement towards pentanal, but still kept some residual activity towards pyruvate. This and the fact that the combination of W57F and R415L (variant M2) leads to an improved acceptance of pentanal support our previous finding that both R415 and W57 are involved in the coordination of the substrate carboxylic group (with W57 being less important) [14]. From this analysis, it was also found that three variants, which were found individually after sequencing, revealed the same (plus additional) mutations: a single amino acid change R415L (variant M1), two amino acid changes R415L/W57F (variant M2) and three amino acid changes R415L/W57F/L417V (variant M3). These variants were used in further studies.

**Table 1:** Analysis of the initial hits after larger scale production and purification ^a^. (*S*)-PBA, (*S*)-1-phenylbutylamine.

Hits	Mutations	U ^b^/mg for (*S*)-PBA:Pyruvate	U/mg for (*S*)-PBA:Pentanal	Improvement over wt for (*S*)-PBA:Pentanal
wt	-	0.86 ± 0.07	0.28 ± 0.03	-
M1	R415L	n.d. ^c^	0.52 ± 0.07	1.85
M2	W57F/R415L	n.d.	0.86 ± 0.002	3.08
M3	W57F/R415L/L417V	n.d.	0.95 ± 0.04	3.41
M4	L56V/R415C	n.d.	0.43 ± 0.01	1.53
M5	L56V/W57F	0.034 ± 0.008	0.31 ± 0.02	1.11
M6	R415F/L417A	n.d.	0.18 ± 0.02	0.63

^a^ For initial determination of the hits, proteins were produced in a 50-mL cultivation scale; cells were disrupted by sonication, and purification was done using manually-packed TALON metal affinity resin columns by gravity flow (buffer compositions can be found in the Experimental Section); ^b^ The reaction was followed at 242 nm for the detection of the product butyrophenone. The assay was conducted using 2.5 mM (*S*)-PBA and 2.5 mM pyruvate/pentanal in 50 mM HEPES buffer pH 7.5 at 30 °C. The activities were calculated as U/mg (purified enzyme). One unit is defined as the formation of 1 µmol of product per minute. Values and standard deviations given are based on three measurements; ^c^ n.d., not detected.

### 2.3. Analysis of the Hits

The VF-ATA variants M1–3 were then produced at a larger scale and purified by affinity chromatography, as well as size exclusion chromatography. Afterwards, all variants and the wild-type were checked again for activity against pyruvate or pentanal and (*S*)-phenylethylamine ((*S*)-PEA), (*S*)-phenylpropylamine ((*S*)-PPA) or (*S*)-phenylbutylamine ((*S*)-PBA) (Table 2). No activity was detected for the three variants when using pyruvate and either (*S*)-PEA or (*S*)-PPA. When using (*S*)-PBA as the amine donor, some residual activity could be determined in the combination with pyruvate, which did not exceed 5% of the wt activity.

In combination with pentanal as amine acceptor, the activity of the VF-ATA could be increased up to five times. The mutation of R415L already had a beneficial effect on the acceptance of longer amine acceptors, like pentanal, presumably as the change of arginine to leucine creates more space in the active site and also increases the hydrophobicity of the pocket, thus increasing the activity of the VF-ATA variants towards more hydrophobic substrates. The addition of the W57F substitution in the active site even increases this effect, especially when using smaller amine donors, like (*S*)-PEA over (*S*)-PPA and (*S*)-PBA. When comparing the acceptance of pentanal with the bulkier amine donors (PPA and PBA), it is observed that there is almost no effect or even a slightly negative effect with the mutation L417V. The change of leucine to valine in the active site seems to decrease the overall activity of the variants.

**Figure 3:**
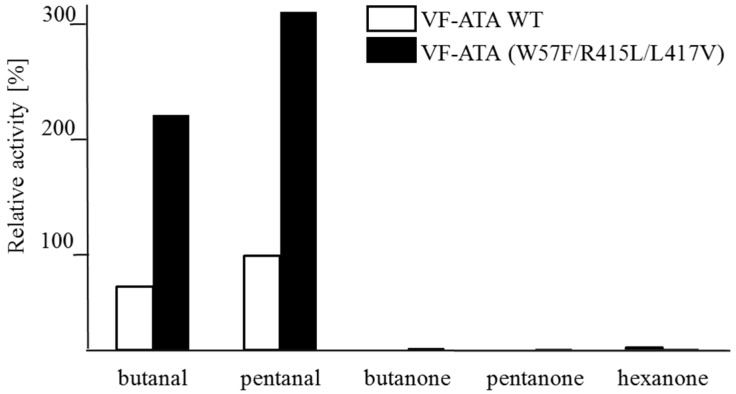
Activity determination using different amine acceptors and (*S*)-PEA as the donor. The wild-type VF-ATA (VF-ATA wt) and the variant M3 (W57F/R415L/L417V)) were verified for the conversion of C4/C5-aldehydes and C4–C6-ketones at pH 9.5. The relative activity was set to 100% pentanal:(*S*)-PEA based on wild-type VF-ATA.

A screening of ketone or aldehyde acceptors with different chain lengths (from C_4_–C_6_) revealed that these protein variants, including the wild-type, prefer aldehydes over ketones (Figure 3). Among the aldehydes, pentanal was accepted better by both the wild-type and the variant compared to butanal. For the VF-ATA, it is already known that the conversion of ketones is limited. It was shown that this ATA has a higher activity when using cyclic ketones, like cyclohexanone over alkyl-ketones [18].

**Table 2:** Determination of the acceptance of other amines by the three top hits ^a^ identified in the library screening. (*S*)-PEA, (*S*)-phenylethylamine; (*S*)-PPA, (*S*)-phenylpropylamine.

Hits ^a^	Mutations	(*S*)-PEA:pyr ^b^	IF ^c^	(*S*)-PPA:pyr	IF	(*S*)-PBA:pyr	IF
wt	-	10.21 ± 0.06	-	2.6 ± 0.2		3.28 ± 0.09	-
M1	R415L	n.d. ^d^	-	n.d.	-	0.118 ± 0.016	**0.04**
M2	W57F/R415L	n.d.	-	n.d	-	0.17 ± 0.02	**0.05**
M3	W57F/R415L/L417V	n.d.	-	n.d.	-	0.080 ± 0.007	**0.02**
**Hits**	**Mutations**	**(*S*)-PEA:pent ^e^**	**IF**	**(*S*)-PPA:pent**	**IF**	**(*S*)-PBA:pent**	**IF**
wt	-	6.17 ± 0.05	-	2.22 ± 0.06	-	1.631 ± 0.097	-
M1	R415L	8.9 ± 0.2	**1.4**	6.35 ± 0.07	**2.9**	4.100 ± 0.007	**2.5**
M2	W57F/R415L	11.04 ± 0.08	**1.8**	9.10 ± 0.09	**4.1**	8.59 ± 0.03	**5.2**
M3	W57F/R415L/L417V	17.08 ± 0.40	**2.8**	6.72 ± 0.07	**3.0**	8.33 ± 0.07	**5.1**

^a^ For a detailed characterization of the hits, proteins were produced in a 200-mL cultivation scale; cells were disrupted by a French press, and purification was done using the FLPC ÄKTA system with metal affinity chromatography followed by size exclusion chromatography (details can be found in the Experimental Section); ^b^ The reaction was followed at 245 nm for the detection of the product acetophenone and at 242 nm for the detection of the products propio- or butyrophenone. The assay was conducted using 2.5 mM amine donor and 2.5 mM pyruvate/pentanal in 50 mM CHES buffer pH 9.5 at 30 °C. The activity ((U/mg) purified enzyme) is displayed by the used donor:acceptor pair. One unit is defined as the formation of 1 µmol of product per minute. Values and standard deviations given are based on at least three measurements. pyr, pyruvate; ^c^ IF, increase factor (wild-type activity was set to 1); ^d^ n.d., not detected; ^e^ pent, pentanal.

The reaction mechanism of ATAs has been studied in great detail [12]. Especially with respect to substrate recognition, transaminases are very versatile enzymes, as they convert chemically-different substrates. This flexibility is realized by the so-called dual substrate mechanism [19]. In VF-ATA, Arg415 is the key residue for recognizing the acidic side chain of the amine acceptor, usually pyruvate, forming a stable salt bridge with its carboxylate group. By changing the arginine residue to leucine, the charged guanidino group of Arg415 is replaced by a shorter hydrophobic and branched side chain. This extends the contact to the amine acceptor and thereby creates space in the active site for a longer acceptor molecule, explaining the increased reactivity of the R415L variants over the wild-type concerning pentanal. Additionally, the active site becomes more hydrophobic, which could be a possible explanation for why pentanal is preferred over butanal. The beneficial effect of W57F concerning the conversion of bulky amines, although found independently here, was already discussed elsewhere [5].

A comparison of the substitutions found in each of the four positions with the ATA sub-dataset from 3DM revealed some interesting points: for arginine 415 (3DM No. 346), it was discovered that there is only one sequence in the whole sub-dataset of amine transaminases (12,956 aligned sequences) where there is a leucine at this position instead of an arginine, like in the wild-type VF-ATA sequence. The discovered sequence is described as amine transferase from *Arthrobacter* sp. (WP_011692948.1.; NC_008541.1.). Interestingly, when checking the whole superfamily of the “ornithine transaminase-like” enzymes (which is not based on the wt VF-ATA sequence), 23 sequences were identified, where a leucine can be found at the position of R415 of VF-ATA (3DM No. 257 in the whole subfamily dataset). Amongst them, there are acetylornithine aminotransferases, adenosylmethionine-8-amino-7-oxononanoate aminotransferase, ornithine/acetylornithine aminotransferase, as well as three putative uncharacterized proteins. For the exchange of leucine 56 to valine (3DM No. 46), there are only two amine transaminase sequences found, one from *Verrucosispora maris* (WP_013734696.1.; NC_015434.1.) and another from *Ketogulonicigenium vulgare* (WP_014537561.1.; NC_0173841.1.). The exchange of tryptophan 57 (3DM No. 47) to phenylalanine can be found in 7.6% of all amine transaminase sequences in the sub-dataset (134 sequences). Concerning leucine 417 (3DM No. 348), a valine can be found in 13.1% of the sequences (in 231 sequences).

### 2.4. pH Profile of VF-ATA Variants M1–3

**Figure 4:**
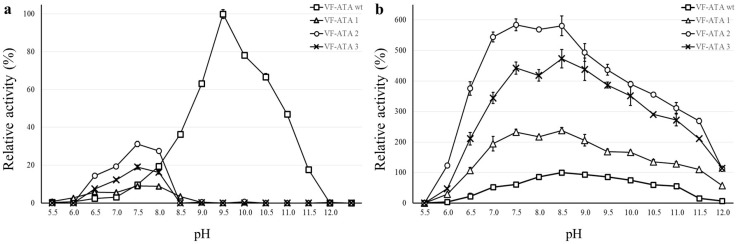
pH profile of the VF-ATA wild-type and variants M1–3. (**a**) pH profile using pyruvate and (*S*)-PEA; (**b**) pH-profile using pentanal and (*S*)-PEA. The relative activity is based on the highest activity of the VF-ATA wild-type in the pH profile, which is at pH 9.5 for pyruvate:(*S*)-PEA and at pH 8.5 for pentanal:(*S*)-PEA. The activity of VF-ATA wild-type is displayed as squares, variant M1 (R415L) as triangles, variant M2 (W57F/R415L) as circles and variant M3 (W57F/R415L/L417V) as crosses.

Characterization of the VF-ATA wild-type and the variants was performed in Davies buffer, as this buffer composition covers a broad pH range from 2 to 12 [20]. Before monitoring the pH range, the activity of all enzyme variants was determined first with standard buffer (50 mM CHES pH 9.5) and compared to Davies buffer (50 mM pH 9.5). Although a decrease in activity could be detected for the Davies buffer system (about 70% of activity compared to CHES), it was used for recording the pH optima. The pH profile of the VF-ATA wild-type and variants M1–3 was monitored independently for pyruvate and pentanal (Figure 4).

With pyruvate as the amine acceptor, the pH optimum of the wild-type VF-ATA was at alkaline pH (pH 9.5). Surprisingly, an increase in activity for the VF-ATA variants M1–3 was observed in the neutral range starting from pH 6.5 to 8.5 with an optimum for all variants at pH 7.5. Here, the VF-ATA variant M2 showed the highest activity of all variants. The activity of VF-ATA M3 is higher than that of variant M1, indicating that the additional mutations to R415L resulted in a beneficial effect for the activity towards pyruvate and (*S*)-PEA at neutral pH (W57F for variant M2 and W57F/L417V for variant M3).

When comparing the activity of the VF-ATA variants towards pentanal and (*S*)-PEA, it was observed that the activity of variant M2 is slightly higher than of variant M3. Both variants have a higher activity compared to variant M1, confirming that at least the W57F mutation results in a beneficial effect for converting aliphatic aldehydes, like pentanal. Due to the higher activity of variant M2 over M3, we assume that the contribution of the L417V mutation to the change of the acceptor/donor spectrum is negligible. However, all variants exhibited an increase in the conversion of aliphatic aldehydes compared to the wild-type VF-ATA. Interestingly, the activity of variant M2 and M3 differed when changing the buffer system. In CHES buffer (pH 9.5), variant M3 had a higher activity (Table 2), whereas in Davies buffer, variant M2 had a slightly higher activity (Figure 4).

All measurements were conducted as soon as possible with fresh protein. For storage, the remaining protein was mixed with glycerol (final concentration 20% *v*/*v*), aliquoted and frozen at −20 °C. The stability of all VF-ATA variants was measured over time using the standard assay conditions with (*S*)-PBA as the amine donor and pyruvate or pentanal as the amine acceptor. The activity of all variants remained stable over a time period of 47 days.

## 3. Experimental Section

### 3.1. Materials

Unless stated otherwise, all chemicals were purchased from Sigma-Aldrich (Steinheim, Germany), Fluka (Buchs, Switzerland), New England Biolabs (Ipswich, MA, USA) or Merck (Darmstadt, Germany). Primers were synthesized by Life Technologies (Darmstadt, Germany). Sequencing was done at Eurofins MWG Operon (Ebersberg, Germany).

### 3.2. Bacterial Strains and Plasmids

*E. coli* TOP10 and *E. coli* BL21 (DE3) were purchased from New England Biolabs (Beverly, MA, USA). The plasmid pET24b containing the gene encoding the (*S*)-ATA from *Vibrio fluvialis* (UniProt: F2XBU9) bearing an additional C-terminal His_6_-tag was kindly provided by Prof. Byung Gee Kim (Seoul National University, Seoul, Korea). Plasmids were routinely transformed into *E. coli* strains by the heat shock method [21].

### 3.3. Generation of the Library Based on the ATA from Vibrio fluvialis

For the generation of the mutant library, degenerate primers containing the desired codons were used (Table 3). The library was created using site-directed mutagenesis of the VF-ATA gene using the QuikChange™ method. A typical PCR mixture (125 µL) consisted of 10× *Pfu* Plus! DNA polymerase buffer (12.5 µL), DMSO (2.5 µL), 2.5 µL of a mixture of deoxynucleoside triphosphates (0.25 mM each), *Pfu* Plus! DNA polymerase (1 U), plasmid DNA (30 ng) and the forward and reverse primers (0.2 µM). After initial denaturation for 2 min at 94 °C, the cycling program was followed for 25 cycles: 30 s, 94 °C denaturation; 30 s, primer annealing at the primer specific optimal annealing temperature; and 8 min, 72 °C elongation. The final elongation step was performed over 16 min at 72 °C. After PCR, the reaction mixtures were digested for 2 h at 37 °C with *Dpn*I, followed by transformation into *E. coli* TOP10. After overnight growth on agar plates supplemented with kanamycin, all clones were washed off from the plate with 1–2 mL LB media, which were used for inoculation of a 5-mL overnight cultivation (LB media, kanamycin complemented). Plasmids were isolated from the overnight culture (innuPREP Plasmid Isolation Kit, Analytik, Jena, Germany). An aliquot of the plasmid preparation was sent to sequencing to verify the quality of the library. The correctness of the library was verified by checking the base distribution at the mutational sites in the sequencing chromatogram. For screening of the library, the isolated plasmids were transformed into *E. coli* BL21 (DE3).

**Table 3:** Overview of the primers used for generating the library. The mutated codons are underlined and highlighted in bold.

Position	Primer Sequences	Mutation	wt
L56A/L/P/V and W57C/L/F/W (forward)	CTCGGGC**SYGTKS**AACATGGTCGCGGGCTTTGACC	SYG and TKS	CTG and TGG
L56A/L/P/V and W57C/L/F/W (reverse)	CCATGTT**SMACRS**GCCCGAGTTGGCGTCCAGATAACGCC	SRC and AMS	GAC and ACC
R415R/C/L/F and L417A/L/P/V (forward)	GATTTGC**YKC**CCG**SYT**GGTCAGTCCGTCGTCC	YKC and SYT	CGG and CTT
R415R/C/L/F and L417A/L/P/V (reverse)	CTGACC**ARS**CGG**GMR**GCAAATCAGCCCCAGATCGGTGC	RMG and SRA	GCC and GAA

### 3.4. Preparation of 96-Well Plates for Screening on the Robotic Platform

Plasmids containing the VF-ATA library, VF-ATA wild-type or the empty pET24b vector were transformed in *E. coli* BL21 (DE3) via the heat shock method. The kanamycin complemented agar plates were incubated at 30 °C overnight. The next morning, the agar plates were transferred to a 37 °C incubator and incubated until the colonies had the appropriate size for the use of the colony picker (QPix 420, Molecular Devices, Sunnyvale, CA, USA). Sterile 96-well microtiter plates were filled with 200 µL/well LB media (kanamycin complemented) and inoculated with the help of the colony picking robot. Sixteen 96-well microtiter plates were prepared, for which each comprised 70 colonies containing the library and as controls, 8 colonies containing the VF-ATA wild-type, 8 colonies containing the empty pET24b plasmid and 10 wells containing only kan-LB medium. The plates were incubated overnight at 37 °C and 600 rpm under a breathable membrane in an automated incubator (Cytomat 2C 1550 LIN ToS, Thermo Fisher Scientific, Waltham, MA, USA). Overnight plate cultures were used to inoculate new sterile 96-well plates filled with 165 µL TB media/well (containing kan) with a 15-µL cell suspension/well. The remaining overnight culture plates were used to prepare a glycerol stock. Therefore, 100 µL/well of glycerol/H_2_O solution (75:25 mixture) were added, and plates were stored in the −80 °C freezer. The freshly-inoculated microtiter plates were used for screening. The high-throughput screening was realized fully automatically on our robotic platform (http://lara.uni-greifswald.de) [16].

### 3.5. Heterologous Protein Production

Materials and media were sterilized by autoclaving or by filtration through 0.20-µm syringe filters. Agar plates were prepared with LB medium supplemented with 1.5% (*w*/*v*) agar. Bacteria on agar plates were incubated in an Incucell Incubator (MMM Medcenter-Einrichtungen GmbH, München, Germany) at 37 °C. For heterologous protein production, all plasmids containing the gene of the wild-type VF-ATA and the target mutants were expressed in *E. coli* BL21 (DE3). Therefore, 200 mL TB media supplied with 0.05 mg/mL kanamycin was inoculated with an overnight culture to give an OD_600_ of 0.05. *E. coli* BL21 (DE3) cells were grown at 37 °C in baffled shake flasks at 37 °C with 200 rpm to an OD_600_ of about 0.6. Expression media in the flasks were cooled to 20 °C, and protein expression was started by adding 0.1 mM isopropyl-β-thiogalactoside IPTG. After 20 h of expression at 20 °C, the cells were harvested by centrifugation at 4350× *g* and 4 °C for 15 min.

### 3.6. Protein Purification

The cell pellets containing the VF-ATA or the variants were resuspended in Buffer A (50 mM HEPES-NaOH pH 7.5, 300 mM sodium chloride), which contained additional 0.1 mM PLP. Cell disruption was performed with one cycle in a French Press at 1500 psi. The cell suspension was centrifuged for 1 h at 10,000× *g*. The filtrated supernatant was applied to a Ni-nitrilo triacetic acid (Ni-NTA) column (GE Healthcare (Buckinghamshire, UK) HiTrap FF 5 mL). After washing the column with two column volumes of 10% Buffer B (Buffer B: 50 mM HEPES-NaOH pH 7.5, 300 mM NaCl, 0.1 mM PLP, 300 mM imidazole) at a flow rate of 5 mL/min, the protein was eluted with 100% Buffer B. Amine transaminase-containing fractions were pooled and afterwards desalted by gel chromatography against 50 mM HEPES-NaOH pH 7.5 containing 0.01 mM PLP at a flow rate of 2 mL/min. The protein content of the purified, desalted fractions was determined via the BCA protein quantification kit (Thermo Scientific, Carlsbad, CA, USA). Standard curves were recorded using BSA in the range of 0.02–2 mg/mL. All samples were measured in triplicate using suitable dilutions.

### 3.7. Activity Measurements

The activity of the VF-ATA and its variants was determined spectroscopically using the (modified) acetophenone-assay [17]. Ten microliters of a 0.2 mg/mL protein dilution were mixed with 90 µL *N*-cyclohexyl-2-aminoethanesulfonic acid (CHES) buffer (50 mM CHES-NaOH, pH 9.5). Pyruvate and pentanal were used as the amine acceptor, and (*S*)-phenylethylamine (PEA), (*S*)-phenylpropylamine (PPA) and (*S*)-phenylbutylamine (PBA) were used as the amine donor. The reaction was started by adding 100 µL activity reaction solution (2.5 mM amine acceptor and 2.5 mM anime donor in CHES-NaOH buffer 50 mM, pH 9.5). The change in absorption was measured at 245 nm (for PEA) or 242 nm (for PPA and PBA) over 30 s at 30 °C. Only the linear slope in the change in absorption was considered for the determination of the specific activity.

## 4. Conclusions

By creating a “small, but smart” library of the amine transaminase from *Vibrio fluvialis*, we were able to shift the acceptor/donor spectrum towards aldehydes bearing a C_4_–C_5_ aliphatic side chain. Four residues of the active site pocket (three hydrophobic: L56, W57, L417; one charged: R415) were selected. With the help of a fully-automatic robotic screening, 1200 variants were investigated resulting in six hits. After the verification of these hits, three variants were found to have the targeted shift in the donor/acceptor spectrum. Sequencing these three hits revealed that they all contain the same R415L mutation plus W57F and L417V. *In silico* analysis of these exchanges using a data subset of the 3DM database containing ~13,000 (putative) amine transaminases of the PLP fold class I reveals that, although some of the exchanges are quite rare, they can be found in other ATA sequences. This study supports the statement that these residues are relevant target points for the change of the substrate spectrum of an amine transaminase.
